# Do Chronic Low Back Pain and Chronic Widespread Pain differ in their association with Depression Symptoms in the 1958 British Cohort?

**DOI:** 10.1093/pm/pnac170

**Published:** 2022-11-04

**Authors:** Cameron Dickson, Ang Zhou, Erin MacIntyre, Elina Hyppönen

**Affiliations:** School of Allied Health Science and Practice, The University of Adelaide, Adelaide, Australia; ACPreH, Unit of Clinical and Health Sciences, University of South Australia, Adelaide, Australia; IIMPACT in Health, Allied Health & Human Performance, University of South Australia, Adelaide, Australia; ACPreH, Unit of Clinical and Health Sciences, University of South Australia, Adelaide, Australia; South Australian Health and Medical Research Institute, Adelaide, South Australia, Australia

**Keywords:** Depression, Low-Back, Widespread, 1958BC, Chronic, Pain

## Abstract

**Objective:**

Depression frequently coexists with chronic pain. Contemporary models suggest that these conditions share pathobiological mechanisms, prompting a need to investigate their temporal association. This investigation aimed to explore two distinctly different chronic pain conditions, and their cross-sectional and prospective associations with depression.

**Methods:**

Self-reported information was available on chronic widespread pain (CWP), chronic low back pain (CLBP) (45 years), and depression symptoms (45 and 50 years) from up to 9,377 participants in the 1958 British cohort. Depression symptom outcomes were derived by “Clinical Interview Schedule-Revised” (45 years) and “Short Form-36” (50 years). Relationships between both chronic pain conditions and depression symptoms were investigated by fitting four separate logistic regression models, each with varying levels of covariate adjustment, including depression at baseline.

**Results:**

CWP was associated with depression symptoms cross-sectionally (odds ratio [OR] = 2.04, 95% confidence interval [CI] 1.65, 2.52; *P *< 0.001, *n *=* *7,629), and prospectively when fully adjusted for baseline, sociodemographic, lifestyle, and health covariates (OR = 1.45, 95% CI 1.17, 1.80; *P *= < 0.001, *n *=* *6,275). CLBP was associated with depression symptoms prospectively (full model: OR = 1.28, 95% CI 1.01, 1.61; *P *= 0.04, *n *=* *6,288). In fully adjusted models the prospective association of CWP with depression symptoms was more heavily influenced by our covariates than CLBP with depression symptoms.

**Conclusion:**

Pain may be a stressor from which depression can arise. Development of depression may be differentially dependant upon the type of pain experienced. Screening for depression symptoms among individuals with both chronic pain conditions is indicated and should be repeated over time.

## Introduction

Chronic pain is prevalent and burdensome, with an estimated one third to half of UK adults experiencing chronic pain [1]. The economic burden of low back pain alone was calculated to be approximately £10 billion per annum in 2000 [2]. Similarly, depression is considered to be the largest contributor to global disability by the World Health Organization [3]. As of 2014 in the United Kingdom, 7.5% of the population was estimated to suffer from depression, with a peak in prevalence observed during middle adulthood [4]. Pain commonly coexists with psychiatric conditions, notably depression, often with devastating individual and societal consequences [5, 6]. Critically, when presenting together depression and chronic pain yield poorer treatment responses and outcomes [7]. Recent research has focused on elucidating the mechanistic basis for the observed coexistence of these two conditions, prompting a need to explore their temporal relationships, particularly regarding potential differences between pain types (e.g., regional and global pain conditions). These relationships are of growing importance to public health policy given that chronic pain prevalence is predicted to rise due to the COVID-19 pandemic [8].

Previous studies have explored the associations between pain and depression cross-sectionally at a population level, and the potentially bidirectional nature of their relationship has been recognised [9]. Chronic pain is a physiological stressor and plausible antecedent to depression [10], yet to our knowledge there are no studies that explore and compare the link between different types of pain conditions and subsequent depression. Regional (e.g., chronic low back pain [CLBP]) and widespread pain (e.g., fibromyalgia) conditions can be considered distinct phenotypes of chronic pain, with observed differences in sensory, clinical, and psychosocial measures [11, 12]. These differences are highlighted in the new IDC-11 classification codes, where chronic widespread pain (CWP) and chronic primary musculoskeletal pain (which includes chronic primary low back pain) are subgroups of chronic primary pain [13]. These subgroups have unique features that differentially overlap with the proposed mechanisms that link chronic pain and depression; therefore, it is important to further understand their independent relationships with depression.

Considerable research has examined the shared biological mechanisms of chronic pain and depression. Both the hypothalamic-pituitary-adrenal axis, and neuroimmune inflammatory mechanisms have been implicated as shared aetiology [14–18]. Recent research has demonstrated upregulated activation of glia in the brain of individuals with fibromyalgia (compared to healthy controls) suggesting that neuroimmune pathways are implicated in CWP conditions [19]. Furthermore, there is evidence of greater neuroimmune activation in the brain of persons with CLBP with radiculopathy (more akin to CWP) compared to those with axial CLBP (a regional pain condition) [20]. This neuroimmune activation was positively correlated with scores from the American College of Rheumatology Fibromyalgia Survey Criteria from each CLBP subgroup, suggesting it may be implicated in widespread symptom profiles, and differ from that of regional symptom profiles [20]. Evidence of differential relationships between pain conditions and depression, will not only provide further evidence for proposed pathobiological mechanisms linking the conditions, but crucially may inform public health policy and treatment pathways such as depression screening protocols in primary healthcare settings, and the provision of integrated health services.

The objectives of this study are to investigate the cross-sectional and prospective associations between CWP and CLBP with depression symptoms in mid-life, using the 45 year (baseline) and 50 year (follow-up) sweeps of the 1958 British Birth Cohort (1958BC, otherwise known as the National Child Development Study). We also aimed to explore how the associations are influenced by socio-demographic, lifestyle, and health factors. We hypothesised that both CWP and CLBP increase the risk of depression symptoms in mid-life. Given the greater overlap of proposed mechanisms between CWP and depression, we hypothesised that these variables would be more closely associated than CLBP and depression, both cross-sectionally and longitudinally.

## Methods

We utilised the 1958BC as it is a rich longitudinal source of health, lifestyle, and sociodemographic data highly relevant to the aims of our investigation, including comprehensive pain and mental health data from participants aged 45 and 50 years (y) [21]. The 1958BC comprised 17,638 individuals born in one week in 1958 across England, Scotland, and Wales, with the subsequent addition of 920 immigrants also born within the same week [22]. Cohort members have been followed since birth with data collection at multiple time points, including a biomedical survey involving objective physical assessment at age 45y [21]. In this study, we used data from surveys at ages 45 and 50y, to construct our baseline (i.e., CLBP, CWP, and depressive symptoms aged 45y) and prospective (i.e., depressive symptoms aged 50y) measures. Data from surveys at ages 33, 42, and 45 were used to provide information on covariates. From the cohort that completed the 45y biomedical survey (*n *=* *9377), the current cross-sectional analyses were restricted to 7629 individuals with information for CWP and depression symptoms (45y), and 7643 individuals with data on CLBP and depression symptoms (45y) (Supplementary Data Figure 1). Prospectively, data were available for 6275 individuals with CWP and depression symptoms (50y); and 6288 individuals with data on CLBP and depression symptoms (50y).

## Depression Symptom Measures

The depression symptom variable (45y) was constructed using a summary score based on the “Clinical Interview Schedule—Revised” (CIS-R) which is a screening instrument for depression, and designed for use by lay persons. In the 1958BC this assessment was conducted using computer assisted personal interview (CAPI) by research nurses [21]. The CIS-R has been previously validated, and comprises multiple sections pertaining to psychiatric symptoms evident within the previous month, which are allocated a numeric score (depression summary score ranging from 0 to 4, based on severity and frequency of symptoms in the preceding week) [23–25]. We constructed a categorical yes/no indicator of depression symptoms (aged 45y) using a cutoff score of ≥2 for the CIS-R, consistent with previous research [23]. From the 1958BC at age 50y data regarding depressive symptoms was available from an alternate assessment tool, the 36-item “Short-Form Health Survey” (SF-36). The SF-36 is a self-completed, paper-based measurement tool yielding information on three primary dimensions: functional status, well-being, and overall evaluation of health, and embodies the Mental Health Index (MHI-5) [26]. The MHI-5 has been validated previously as a means of screening for symptoms of depression and provides scores ranging from 0 to 100 [27]. Again, a categorical yes/no indicator of depression symptoms was constructed using cut-off scores consistent with prior research (≤52 indicating depressive symptoms) [28].

## Chronic Widespread Pain and Chronic Low Back Pain Measures

CWP and CLBP were each obtained from the biomedical survey via a construct of self-reported pain related information [21]. Questions pertained to the duration of painful symptoms (delineating acute vs. chronic pain, defined here as pain lasting longer than three months), and a body chart on which the cohort member was required to shade in the painful regions [29]. From this, multiple pain location variables were derived enabling construction of the CWP variable, such that it aligned with the following subjective pain related component of fibromyalgia 1990 diagnostic criteria: “pain lasting more than three months that is bilateral, axial, and present above and below the waist” [30]. Specifically, our CWP measure included individuals with painful symptoms in their lower body (i.e., below the gluteal folds), trunk and/or upper limb regions. The CLBP variable was constructed in keeping with the following definition: “Low back pain is pain, muscle tension, or stiffness localized below the costal margin and above the inferior gluteal folds” and was considered chronic when persisting for longer than 12 weeks [31]. Symptoms extending into the legs were excluded from our CLBP measure, as were individuals meeting our aforementioned CWP criteria. In doing so, our approach ensured that the two pain cohorts were distinct from each other, with no overlapping cases.

## Sociodemographic, Lifestyle, and Health Covariates

The rationale for the selection of covariates was based on existing evidence of association with the respective exposures (CWP and CLBP) and the outcome (depression symptoms) [32]. Covariate measures were derived from self-completed questionnaires aged 45y (i.e., general health, screen time for leisure, social contact, active social group membership, bill payment difficulty, and separation status). Computer assisted self-interview (i.e., for alcohol intake aged 45y), computer assisted personal interview (i.e., for smoking status and physical activity aged 45y, social class aged 42y), and nurse-led physical testing (i.e., weight and height aged 45y) were also sources of covariate information. Social class was based on the Registrar General’s classification: professional and managerial/technical, skilled non-manual and manual, and unskilled and other/unknown [33]. The highest qualification was constructed using maximum qualifications aged 33y (derived variable from interview) and complemented with information on lifelong learning from the 42y survey [21]. This categorization included nil qualifications, secondary, and tertiary, and was consistent with prior research. Separation status was categorised into yes/no, and bill payment difficulty into minor/no difficulty, difficulty, and great difficulty. Social contact (regularity of contact with friends or acquaintances) was categorised as multiple times per month, better reflecting data distribution and reducing modelling parameters [34]. Active social group membership (i.e., a member of a social/recreational group, trade unions, commercial/professional organisations, political/advocacy/cultural or sporting group) was categorised as yes/no. Screen-time for leisure was constructed combining PC and TV utilization, and categorised as non-sedentary (≤ 4 hours) and sedentary (> 4 hours). Alcohol intake was categorised as never/rarely, light/moderate, and heavy consumption, while general health was either excellent, good, or fair. Body Mass Index (BMI) was calculated as weight (kg)/height (m)^2^ aged 45y. BMI categorisation was based on the World Health Organization classification [35], and included underweight (<18.5), normal (18.5 to <25), overweight (25 to <30), and obese (≥30). Physical activity was constructed using three related measures, regular exercise participation, exercise frequency, and physical demands of work. Categories include four times per month or more and three times per month or less, to better reflect data distribution. Smoking categories include current, ex/occasional, and never.

## Statistical Analyses

We explored descriptive distributions by tabulating frequencies and χ^2^ testing to compare bivariate relationships. Relationships of the chronic pain conditions with depression symptoms were investigated by fitting four separate logistic regression models, each with a different level of covariate adjustment guided by a backward stepwise modelling approach [36]. For cross-sectional analyses of CWP and CLBP associations with depression symptoms (45y), the four levels of adjustment were as follows: (1) Baseline: sex; (2) Socio-demographic: sex, social class, highest education level, separation status, bill payment difficulty; (3) Lifestyle: sex, smoking status, physical activity, alcohol intake, social contact, active member of a social group; (4) Health: sex, and general health. Similar covariate adjustment was used for prospective associations of CWP and CLBP with depression symptoms (50 years), with physical activity being replaced by sedentary status and all models additionally adjusted for baseline depression symptoms and BMI. Complete case analysis was used throughout our analyses. As sensitivity analyses, we imputed missing covariate data using using 20 imputed datasets, and re-iterated all the analyses [37]. All analyses were conducted in STATA 14 (StataCorp LP, Texas USA).

## Results

Our sample included 8,522 individuals with data available for CWP and depressive symptoms, and 8,539 individuals with data available for CLBP and depressive symptoms, both at age 45y. Of the first group, 1,050 (12.3%) of individuals reported CWP at age 45y. Depression rates in this group were stable over time, with 18.5% of individuals having depressive symptoms at 45y, and 18.6% at 50 years. For the CLBP and depression group, 1020 (11.9%) reported CLBP at 45y. Of this group, 7.9% experienced depression at 45y, which increased to 15% at age 50y. Supplementary Data Figure 1 depicts the participant flow through the study. Our descriptive analyses further demonstrated that the prevalence of CWP was associated with lower social class, lower education level, higher bill payment difficulty, smoking, reduced physical activity, increased screen time, lower alcohol intake, not being an active social group member, poor general health, and higher BMI (Supplementary Data Table 1). CLBP was associated with lower education level, smoking, and poor general health (Table 1, Supplementary Data Table 1). Depressive symptoms at ages 45 and 50y were more prevalent in women than in men (Table 1, Supplementary Data Table 1). Lower education level, lower social class, higher bill payment difficulty, smoking, reduced physical activity, reduced social contact, not being an active social group member, increased screen time, lower alcohol intake, and poor general health were all associated with depressive symptoms aged 45 and 50y (Table 1, Supplementary Data Table 1).

**Table 1.** Characteristics of the 1958BC sample, and prevalence in the chronic pain and depression conditions

	N (%)*	CWP 45 (%)	CLBP 45 (%)	DEP 45 (%)	DEP 50 (%)
Gender					
Male	4665 (49.75)	10.5	11.4	7.1	8.1
Female	4712 (50.25)	12.0	10.5	9.7	11.6
Social class					
Professional and managerial/technical	3776 (40.27)	8.1	10.8	6.2	7.7
Skilled non-manual & manual	4848 (51.70)	12.6	11.0	8.7	10.7
Unskilled and other/unknown	753 (8.03)	18.2	10.6	17.7	15.7
Smoking status					
Never	4156 (44.32)	9.4	9.9	7.3	8.7
Ex/occasional smoker	2721 (29.02)	11.8	11.4	7.4	9.2
Smoker	2202 (23.48)	14.1	12.2	11.4	12.9
Missing^†^	298 (3.18)	3.1	3.5	4.6	2.9
General health					
Excellent	1551 (16.54)	3.7	7.2	3.2	5.3
Good	5810 (61.96)	9.5	11.5	6.5	7.9
Fair/poor	1754 (18.71)	24.7	13.2	19.0	20.5
Missing^†^	262 (2.79)	1.5	1.1	3.4	2.5
Body Mass Index					
Normal	3116 (33.23)	10.0	10.7	8.3	7.6
Underweight	51 (0.54)	13.6	9.1	17.6	20.5
Overweight	3796 (40.48)	12.0	12.8	7.4	6.9
Obese	2247 (23.96)	15.9	12.3	10.0	9.1
Missing^†^	167 (1.78)	18.2	14.3	11.9	11.6

**CWP 45** = Chronic widespread pain cases aged 45 years; **CLBP 45** = Chronic low back pain cases aged 45 years; **DEP 45** = Depression aged 45 years; **DEP 50** = Depression aged 50 years.

* **N **=** **9,377.

† Indicates percentage of phenotype missing information.

CWP was associated with depression symptoms in cross-sectional analysis aged 45 years, (odds ratio [OR] = 3.15, 95% confidence interval [CI] 2.59, 3.84; *n *=* *7,629) (Table 2). Association between CWP and depression symptoms was attenuated with each layer of covariate adjustment (socio-demographic, lifestyle, and health). However, even in fully adjusted models, participants with CWP had two-fold higher odds of depression symptoms compared to others (OR = 2.04, 95% CI 1.65, 2.52; *n *=* *7,629). Fully adjusted sensitivity analysis using multiple imputation of missing covariates yielded similar results (OR = 2.04, 95% CI 1.68, 2.48; *n *=* *8,522).

**Table 2.** Associations of chronic pain conditions aged 45 years with depression aged 45 and 50 years

		Adjusted for Sex OR (95% CI)	Adjusted for Sex and Sociodemographic OR (95% CI)	Adjusted for Sex and Lifestyle OR (95% CI)	Adjusted for Sex and Health OR (95% CI)	Full Model OR (95% CI)	Sensitivity Analysis^§^ OR (95% CI)
CWP	Cross-sectional*						
	N = 7,629^‡^	3.15 (2.59 – 3.84)	2.66 (2.17 – 3.26)	2.89 (2.36 – 3.53)	2.19 (1.77 – 2.69)	2.04 (1.65 – 2.52)	2.04 (1.68 – 2.48)
	*P* value	<.001	<.001	<.001	<.001	<.001	<.001
	Prospective^†^						
	N = 6,275^‡^	2.17 (1.78 – 2.65)	1.81 (1.47 – 2.22)	1.99 (1.63 – 2.44)	1.62 (1.32 – 2.00)	1.45 (1.17 – 1.80)	1.44 (1.18 – 1.77)
	*P* value	<.001	<.001	<.001	<.001	<.001	<.001
CLBP	Cross-sectional*						
	N = 7,643^‡^	1.10 (0.85 – 1.42)	1.04 (0.80 – 1.35)	1.07 (0.83 – 1.38)	0.97 (0.75 – 1.26)	0.97 (0.75 – 1.27)	0.89 (0.69 – 1.14)
	*P* value	.47	.75	.62	.85	.85	.36
	Prospective^†^						
	N = 6,288^‡^	1.39 (1.11 – 1.74)	1.33 (1.06 – 1.68)	1.36 (1.08 – 1.70)	1.30 (1.03 – 1.63)	1.28 (1.01 – 1.61)	1.22 (0.98 – 1.52)
	*P* value	<.001	.01	.01	.03	.04	.08

CWP = chronic widespread pain; CLBP = chronic low back pain.

* Adjustment of logistic regression models in cross-sectional analyses—Baseline: sex; Sociodemographic: sex, social class, highest education level, separation status, bill payment difficulty; Lifestyle: sex, smoking status, physical activity, alcohol intake, social contact, active member of a social group; Health: sex, and general health.

† Adjustment of logistic regression models in prospective analyses—**Baseline:** sex and depression; **Socio-demographic:** sex, depression (45 years), social class, highest education level, bill payment. difficulty; **Lifestyle:** sex, depression (45 years), smoking status, alcohol intake, social contact active member of a social group, sedentary status; **Health:** sex, depression (45 years), BMI, and general health.

‡ N = Adjusted in full (all models nested).

§ **Sensitivity analyses** (completed by multiple imputation of missing covariates in the full model—using 20 imputed datasets) - **Cross-sectional:** CWP N = 8,522, CLBP N = 8,539, **Prospective:** CWP N = 6,928, CLBP N = 6,943.

CWP was also associated with depression symptoms (50 years) in prospective analyses, which controlled for depression symptoms at baseline (OR = 2.17, 95% CI 1.78, 2.65; *n *=* *6,275) (Table 2). Adjustment for sociodemographic, lifestyle, and health related covariates each attenuated, but did not fully explain, the prospective association between CWP and depression symptoms (OR = 1.45, 95% CI 1.17, 1.80; *n *=* *6,275). Fully adjusted sensitivity analysis using multiple imputation provided very similar results (OR = 1.44, 95% CI 1.18, 1.77; *n *=* *6,943).

CLBP was not associated with depression symptoms at baseline (age 45 y) (fully adjusted OR = 0.97, 95% CI 0.75, 1.27; *n *=* *7,643) (Table 2). Fully adjusted sensitivity analyses using multiple imputation on missing values similarly evidenced no association (OR = 0.89, 95% CI 0.69, 1.14; *n *=* *8,539).

In prospective analyses, CLBP (45 years) was associated with depression symptoms (50 years) (OR = 1.39, 95% CI 1.11, 1.74; *n *=* *6,288); an association which was partly attenuated by full adjustment (OR = 1.28, 95% CI 1.01, 1.61; *n *=* *6,288) (Table 2). Again, sensitivity analyses using multiple imputation were similar, though with greater imprecision (OR = 1.22, 95% CI 0.98, 1.52; *n *=* *6,958).

## Discussion

To our knowledge this is the first study investigating the association between chronic pain and depression symptoms in mid-life, within the 1958BC. We found that CWP was strongly associated with depression symptoms both cross-sectionally and prospectively. This association was maintained when controlling for socio-demographic, lifestyle, and health covariates. In contrast, CLBP was only prospectively associated with depression symptoms. While both chronic pain conditions were prospectively associated with depression symptoms in the fully adjusted models, CWP was more heavily influenced by covariate adjustments than CLBP. That CWP was associated both cross-sectionally and prospectively with depression symptoms suggests they may share common pathobiological mechanisms.

The cross-sectional association between CWP and depression symptoms is consistent with previous research [38]. In our cohort, people with CWP at age 45 had a 45% higher risk of developing depression symptoms by 50y. This is lower than a previous study, which found that people with fibromyalgia (*n *=* *25,969) are over seven times more likely to be diagnosed with depression in the following 3–11 years [39]. The lower risk in the current study may be due to differences between CWP and fibromyalgia. Diagnosis of fibromyalgia requires not only widespread pain, but also additional somatic symptoms (e.g., fatigue, abdominal symptoms) [40]. Moreover, previous research has demonstrated higher levels of psychological distress in persons with meeting the diagnostic criteria for fibromyalgia, compared to those with CWP alone [41]. Finally, the shorter follow-up period of this study may influence effect sizes. The present study found no cross-sectional association between CLBP and depression aged 45y. This is in contrast with a cross-sectional Canadian study (*n *=* *118,000) in which people with CLBP were 6.2 times more likely to experience depression than those without pain [42]. Further, in the United Kingdom Keeley et al. (2008) found 25% of CLBP (*n *=* *108) patients suffered from comorbid depressive symptoms [43]. Interestingly, both studies used a time frame of 6 months for pain chronicity (compared to 3 months in this study). The differences in inclusion criteria may explain the inconsistent results, given that that changes in pain levels over a 3-month period can predict changes in depression outcomes [44]. Nevertheless, our prospective analysis implicates CLBP as a predictor of subsequent depression symptoms, which is consistent with previous research efforts [45]. Additionally, we were able to control for a number of sociodemographic, lifestyle, and health covariates in our analysis; these factors attenuated, but did not fully explain, the relationships between the two pain conditions and depression symptoms.

Multiple models of the chronic pain and depression relationship exist in the literature, many indicating that the two conditions have shared pathobiological mechanisms [10]. Considerable evidence from prior research supports dysregulation of the hypothalamic-pituitary-adrenal axis as a plausible biological pathway linking chronic pain and depression [18]. Similarly, recent research suggests that both pain and psychological stress can initiate a cascade of cellular reactions whereby neuro-immune (i.e., microglial) mechanisms are upregulated, leading to depression [46]. Our results support a model in which chronic pain is a stressor from which depression can develop, which culminates differently depending on the nature of the pain experience (i.e., regional compared to systemic). Stress response systems seem a likely pathway moderating the relationship of these conditions.

The results of this study have significant clinical implications. The longitudinal relationship between chronic pain and depression indicates that repeated screening for depression is required. This is particularly important for regional pain conditions (such as CLBP), as depression may develop over time; if screening only occurs at diagnosis, subsequent development of depression could be missed. Additionally, the strong association between pain conditions and depression, as well as that people experiencing both pain and depression have an attenuated response to treatment, indicates that this group may merit special consideration with regards to treatment pathways—early referral to multi-disciplinary care may be appropriate for this population. Socio-demographic, lifestyle and health covariates had a considerable influence on the prospective relationship between CWP and depression. This highlights the importance of such factors in influencing the health outcomes in people with CWP.

### Limitations

As with all observational studies, despite comprehensive covariate adjustment we are unable to discount effects by residual confounding, and therefore associations observed in our study may not be causal. Secondly, while in line with previously validated methodologies [47, 48], there are also limitations with the construction of both chronic pain measures. Namely, we did not integrate pain intensity, higher levels of which have previously been associated with greater depression symptoms [49], which may have affected our findings. Furthermore, chronic pain has known associations with other psychiatric conditions (e.g., post-traumatic stress disorder) which may confound our findings. Presence of multiple psychiatric conditions may be a confounder [50]. Our CLBP measure excluded individuals with symptoms extending to their legs. This was done to ensure that the CLBP and CWP conditions were phenotypically distinct, and to exclude potential cases of lumbo-genic radiculopathy, as neuropathic pain typically has a greater association with adverse mental health than somatic chronic pain alone [51]. We sought to mitigate the influence of neuropathic pain as a potential confounder for CLBP, though we were unable to do this for CWP. This may contribute to the weaker associations between CLBP and depression symptoms. Also, the study design did not account for comorbid musculo-skeletal complaints such as neck pain or other comorbid multi-joint pain, which commonly co-exists with both low back pain and depression [52, 53]. Regarding CWP, our measure closely aligns with the diagnostic criteria for fibromyalgia but does not include other symptoms required for diagnosis (e.g., measures of fatigue, cognitive issues). Caution is needed when aligning the CWP results with fibromyalgia, as the CWP variable constructed here may also be comparable to other conditions where widespread pain is a feature, for example auto-immune polyarthropathies [54]. While our results support a temporal relationship of chronic pain and downstream development of depression, our study does not account for the possibility of depression preceding the onset of pain and predisposing individuals to subsequent depression [10]. Finally, the depression symptoms variable at the two timepoints were constructed using different measures. However, both measures have been validated. The CIS-R has been validated against an interviewer-led version of the same for depression [55], while the MHI-5 has been validated for detecting mood disorders and psychological distress in various populations [28, 56].

In conclusion, both CWP and CLBP appear to have a temporal and differential relationship with the latter onset of depression, where CWP is associated with depression symptoms cross-sectionally and prospectively; and CLBP is only associated with depression symptoms prospectively. Concomitance between CWP and affective mechanisms is suggested. Health, lifestyle, and socio-demographic factors were observed to influence the associations of both pain conditions with depression. Our results have implications for clinical management of individuals with chronic pain. Considerable attention to a chronic pain sufferer’s psychological wellbeing is needed, and formalised screening for depression symptoms among this demographic may be indicated for best practice. Given the temporal relationship, this screening should be periodically repeated over time. Moreover, given that the chronic pain and depression frequently co-occur, dedicated multi-disciplinary treatment pathways should be developed to allow co-treatment of both conditions.

## Authors’ Contributions

All listed authors have made substantial contributions to the conception and design of the study, and/or data acquisition, analysis and interpretation of data, drafting of the manuscript, and final approval of the submission.

## Supplementary Material

pnac170_Supplementary_Data

## Notes

Funding sources: This study was completed within a research candidature (CD) funded by the Australian Government Research Training Scholarship. The funders played no role in the design, conduct, or reporting of this study.

## References

[1] Fayaz A , Croft P, Langford RM, Donaldson LJ, Jones GT. Prevalence of chronic pain in the UK: A systematic review and meta-analysis of population studies. BMJ Open 2016;6(6):e010364. 10.1136/bmjopen-2015-010364 PMC4932255 27324708

[2] Maniadakis N , Gray A. The economic burden of back pain in the UK. Pain 2000;84(1):95–103. 10601677 10.1016/S0304-3959(99)00187-6

[3] WHO. Depression and Other Common Mental Disorders Global Health Estimates. Geneva, SUI: WHO, 2017.

[4] Arias de la Torre J , Vilagut G, Ronaldson A, et al Prevalence and age patterns of depression in the United Kingdom: A population-based study. J Affect Disord 2021;279:164–72. 33059219 10.1016/j.jad.2020.09.129

[5] Ghavidel-Parsa B , Bidari A, Amir Maafi A, Ghalebaghi B. The iceberg nature of fibromyalgia burden: The clinical and economic aspects. Kor J Pain 2015;28(3):169–76. 10.3344/kjp.2015.28.3.169 PMC4500780 26175876

[6] Martini L , Hoffmann F. Comorbidity of chronic back pain and depression in Germany: Results from the GEDA study, 2009 and 2010. Zeitschrift Fur Evidenz, Fortbildung Und Qualitat im Gesundheitswesen 2018;137-138:62–8. 30415830 10.1016/j.zefq.2018.10.003

[7] Arnow BA , Hunkeler EM, Blasey CM, et al Comorbid depression, chronic pain, and disability in primary care. Psychosom Med 2006;68(2):262–8. 16554392 10.1097/01.psy.0000204851.15499.fc

[8] Clauw DJ , Häuser W, Cohen SP, Fitzcharles M-A. Considering the potential for an increase in chronic pain after the COVID-19 pandemic. Pain 2020;161(8):1694–7. 32701829 10.1097/j.pain.0000000000001950 PMC7302093

[9] Bondesson EL , Pardo F, Stigmar K, et al Comorbidity between pain and mental illness – Evidence of a bidirectional relationship. Eur J Pain 2018;22(7):1304–11. 10.1002/ejp.1218.) 29577509

[10] Fishbain DA , Cutler R, Rosomoff HL, Rosomoff RS. Chronic pain-associated depression: Antecedent or consequence of chronic pain? A review. Clin J Pain 1997;13(2):116–37. 9186019 10.1097/00002508-199706000-00006

[11] Gerhardt A , Eich W, Janke S, Leisner S, Treede RD, Tesarz J. Chronic widespread back pain is distinct from chronic local back pain: Evidence from quantitative sensory testing, pain drawings, and psychometrics. Clin J Pain 2016;32(7):568–79. 26379077 10.1097/AJP.0000000000000300

[12] Finnern MM , Kleinböhl D, Flor H, Benrath J, Hölzl R. Differential sensory and clinical phenotypes of patients with chronic widespread and regional musculoskeletal pain. Pain 2021;162(1):56–70. 32773595 10.1097/j.pain.0000000000002018

[13] Nicholas M , Vlaeyen JWS, Rief W, et al; IASP Taskforce for the Classification of Chronic Pain. The IASP classification of chronic pain for ICD-11: Chronic primary pain. Pain 2019;160(1):28–37. 30586068 10.1097/j.pain.0000000000001390

[14] Gosselin R-D , Suter MR, Ji R-R, Decosterd I. Glial cells and chronic pain. Neuroscientist 2010;16(5):519–31. 20581331 10.1177/1073858409360822 PMC3017463

[15] Ji RR , Berta T, Nedergaard M. Glia and pain: Is chronic pain a gliopathy? Pain 2013;154 Suppl 1:(S10–s28. 23792284 10.1016/j.pain.2013.06.022 PMC3858488

[16] Singhal G , Baune BT. Microglia: An interface between the loss of neuroplasticity and depression. Front Cell Neurosci 2017;11:270. 28943841 10.3389/fncel.2017.00270 PMC5596091

[17] McBeth J , Chiu YH, Silman AJ, et al Hypothalamic-pituitary-adrenal stress axis function and the relationship with chronic widespread pain and its antecedents. Arthritis Res Ther 2005;7(5):R992–1000. 16207340 10.1186/ar1772 PMC1257426

[18] Vreeburg SA , Hoogendijk WJG, van Pelt J, et al Major depressive disorder and hypothalamic-pituitary-adrenal axis activity: Results from a large cohort study. Arch Gener Psychiatry 2009;66(6):617–26. 10.1001/archgenpsychiatry.2009.50 19487626

[19] Albrecht DS , Forsberg A, Sandström A, et al Brain glial activation in fibromyalgia – a multi-site positron emission tomography investigation. Brain Behav Immun 2019;75:72–83. 30223011 10.1016/j.bbi.2018.09.018 PMC6541932

[20] Alshelh Z , Brusaferri L, Saha A, et al Neuroimmune signatures in chronic low back pain subtypes. Brain 2022;145(3):1098–110. 34528069 10.1093/brain/awab336 PMC9128369

[21] Power C , Elliott J. Cohort profile: 1958 British birth cohort (National Child Development Study). Int J Epidemiol 2006;35(1):34–41. 16155052 10.1093/ije/dyi183

[22] Atherton K , Fuller E, Shepherd P, Strachan DP, Power C. Loss and representativeness in a biomedical survey at age 45 years: 1958 British birth cohort. J Epidemiol Commun Health 2008;62(3):216–23. 10.1136/jech.2006.058966 18272736

[23] Das-Munshi J , Goldberg D, Bebbington PE, et al Public health significance of mixed anxiety and depression: Beyond current classification. Br J Psychiatry 2008;192(3):171–7. 18310574 10.1192/bjp.bp.107.036707

[24] Botega NJ , Pereira WA, Bio MRG, Junior C, Zomignani MA. Psychiatric morbidity among medical in-patients: A standardized assessment (GHQ-12 and CIS-R) using ‘lay’ interviewers in a Brazilian hospital. Soc Psychiatry Psychiatr Epidemiol 1995;30(3):127–31. 7624806 10.1007/BF00802041

[25] Lewis G , Pelosi AJ, Araya R, Dunn G. Measuring psychiatric disorder in the community: A standardized assessment for use by lay interviewers. Psychol Med 1992;22(2):465–86. 1615114 10.1017/s0033291700030415

[26] Brazier JE , Harper R, Jones NM, et al Validating the SF-36 health survey questionnaire: New outcome measure for primary care. BMJ 1992;305(6846):160–4. 1285753 10.1136/bmj.305.6846.160 PMC1883187

[27] Winston M , Smith J. A trans-cultural comparison of four psychiatric case-finding instruments in a Welsh community. Soc Psychiatry Psychiatr Epidemiol 2000;35(12):569–75. 11213847 10.1007/s001270050281

[28] Strand BH , Dalgard OS, Tambs K, Rognerud M. Measuring the mental health status of the Norwegian population: A comparison of the instruments SCL-25, SCL-10, SCL-5 and MHI-5 (SF-36). Nord J Psychiatry 2003;57(2):113–8. 12745773 10.1080/08039480310000932

[29] Treede R-D , Rief W, Barke A, et al A classification of chronic pain for ICD-11. Pain 2015;156(6):1003–7. 25844555 10.1097/j.pain.0000000000000160 PMC4450869

[30] Wolfe F , Smythe HA, Yunus MB, et al The American College of Rheumatology 1990 Criteria for the Classification of Fibromyalgia. Report of the Multicenter Criteria Committee. Arthritis and Rheumatism 1990;33(2):160–72. 2306288 10.1002/art.1780330203

[31] Chou R. Low back pain (chronic). BMJ Clin Evid 2010;2010:1116. PMC3217809 21418678

[32] VanderWeele TJ , Shpitser I. On the definition of a confounder. Ann Stat 2013;41(1):196–220. 25544784 10.1214/12-aos1058 PMC4276366

[33] Rose D , Pevalin D. The National Statistics Socioeconomic Classification: Unifying Official and Sociological Approaches to the Conceptualisation and Measurement of Social Class. Colchester: University of Essex; 2001.

[34] Agresti A. Categorical Data Analysis. 3rd edition. Hoboken, NJ: John Wiley & Sons; 2013.

[35] WHO. Obesity and Overweight. Geneva, SUI: World Health Organization; 2018.

[36] McDonald JH. Handbook of Biological Statistics. Baltimore, MD: Sparky House Publishing; 2009.

[37] Graham JW , Olchowski AE, Gilreath TD. How many imputations are really needed? Some practical clarifications of multiple imputation theory. Prev Sci 2007;8(3):206–13. 17549635 10.1007/s11121-007-0070-9

[38] Benjamin S , Morris S, McBeth J, Macfarlane GJ, Silman AJ. The association between chronic widespread pain and mental disorder: A population-based study. Arthritis Rheum 2000;43(3):561–7. 10728749 10.1002/1529-0131(200003)43:3<561::AID-ANR12>3.0.CO;2-O

[39] Chang M-H , Hsu J-W, Huang K-L, et al Bidirectional association between depression and fibromyalgia syndrome: A nationwide longitudinal study. J Pain 2015;16(9):895–902. 26117813 10.1016/j.jpain.2015.06.004

[40] Wolfe F , Häuser W. Fibromyalgia diagnosis and diagnostic criteria. Ann Med 2011;43(7):495–502. 21770697 10.3109/07853890.2011.595734

[41] White KP , Nielson WR, Harth M, Ostbye T, Speechley M. Chronic widespread musculoskeletal pain with or without fibromyalgia: Psychological distress in a representative community adult sample. J Rheumatol 2002;29(3):588–94. 11908578

[42] Currie SR , Wang J. Chronic back pain and major depression in the general Canadian population. Pain 2004;107(1-2):54–60. 14715389 10.1016/j.pain.2003.09.015

[43] Keeley P , Creed F, Tomenson B, Todd C, Borglin G, Dickens C. Psychosocial predictors of health-related quality of life and health service utilisation in people with chronic low back pain. PAIN^®^ 2008;135(1-2):142–50. 17611036 10.1016/j.pain.2007.05.015

[44] Kroenke K , Wu J, Bair MJ, Krebs EE, Damush TM, Tu W. Reciprocal relationship between pain and depression: A 12-month longitudinal analysis in primary care. J Pain 2011;12(9):964–73. 21680251 10.1016/j.jpain.2011.03.003 PMC3222454

[45] Meyer T , Cooper J, Raspe H. Disabling low back pain and depressive symptoms in the community-dwelling elderly: A prospective study. Spine 2007;32(21):2380–6. 17906583 10.1097/BRS.0b013e3181557955

[46] Ji R-R , Berta T, Nedergaard M. Glia and pain: Is chronic pain a gliopathy? PAIN^®^ 2013;154(suppl 1):S10–S28. 23792284 10.1016/j.pain.2013.06.022 PMC3858488

[47] Southerst D , Côté P, Stupar M, Stern P, Mior S. The reliability of body pain diagrams in the quantitative measurement of pain distribution and location in patients with musculoskeletal pain: A systematic review. J Manipulative Physiol Ther 2013;36(7):450–9. 23845196 10.1016/j.jmpt.2013.05.021

[48] Southerst D , Stupar M, Côté P, Mior S, Stern P. The reliability of measuring pain distribution and location using body pain diagrams in patients with acute whiplash-associated disorders. J Manipulative Physiol Ther 2013;36(7):395–402. 23891480 10.1016/j.jmpt.2013.05.023

[49] Glombiewski JA , Hartwich-Tersek J, Rief W. Depression in chronic back pain patients: Prediction of pain intensity and pain disability in cognitive-behavioral treatment. Psychosomatics 2010;51(2):130–6. 20332288 10.1176/appi.psy.51.2.130

[50] Fishbain DA , Pulikal A, Lewis JE, Gao J. Chronic pain types differ in their reported prevalence of Post-Traumatic Stress Disorder (PTSD) and there is consistent evidence that chronic pain is associated with PTSD: An evidence-based structured systematic review. Pain Med 2017;18(4):711–35. 27188666 10.1093/pm/pnw065

[51] Inoue S , Taguchi T, Yamashita T, Nakamura M, Ushida T. The prevalence and impact of chronic neuropathic pain on daily and social life: A nationwide study in a Japanese population. Eur J Pain (London, England) 2017;21(4):727–37. 10.1002/ejp.977 PMC5363338 28107599

[52] Mäntyselkä P , Lupsakko T, Kautiainen H, Vanhala M. Neck-shoulder pain and depressive symptoms: A cohort study with a 7-year follow-up. Eur J Pain 2010;14(2):189–93. 19410490 10.1016/j.ejpain.2009.04.004

[53] Gandhi R , Zywiel MG, Mahomed NN, Perruccio AV. Depression and the overall burden of painful joints: An examination among individuals undergoing hip and knee replacement for osteoarthritis. Arthritis 2015;2015:327161. 25861476 10.1155/2015/327161 PMC4377445

[54] Häuser W , Perrot S, Sommer C, Shir Y, Fitzcharles M-A. Diagnostic confounders of chronic widespread pain: Not always fibromyalgia. Pain Rep 2017;2(3):e598. 29392213 10.1097/PR9.0000000000000598 PMC5741304

[55] Head J , Stansfeld SA, Ebmeier KP, et al Use of self-administered instruments to assess psychiatric disorders in older people: Validity of the General Health Questionnaire, the Center for Epidemiologic Studies Depression Scale and the self-completion version of the revised Clinical Interview Schedule. Psychol Med 2013;43(12):2649–56. 23507136 10.1017/S0033291713000342 PMC3821376

[56] McCabe CJ , Thomas KJ, Brazier JE, Coleman P. Measuring the mental health status of a population: A comparison of the GHQ–12 and the SF–36 (MHI–5). Br J Psychiatry 1996;169(4):517–521. 10.1192/bjp.169.4.516 8894205

