# Supplementary material for: Do Chronic Low Back Pain and Chronic Widespread Pain differ in their association with Depression Symptoms in the 1958 British Cohort?
Source: Pain Med. 2022 Nov 4;24(6):644–51. doi: 10.1093/pm/pnac170 (PMC10233498; doi:10.1093/pm/pnac170)
Supplement: pnac170_Supplementary_Data [file pnac170_supplementary_data.zip › pnac170_Supplementary_Data/Supplementary Figure 1.docx]

**1958 British Birth Cohort original sample = 18,558^a^**

**1,245 deceased**

**1,300 emigrated**

**3,004 lost contact**

**1,038 permanent refusal**

**Biomedical survey (45y) target = 11,971^a^**

**2,594 non-respondents**

**Biomedical survey (45y) respondents = 9,377^a^**

**838 missing data CLBP & Dep (45y)**

**855 missing data CWP & Dep (45y)**

**CLBP (45y) & Dep (45y) cohort = 8539**

**CWP (45y) & Dep (45y) cohort = 8522**

**896 missing covariate data**

**893 missing covariate data**

**Cross-sectional ^c^ full model = 7643**

**Cross-sectional ^b^ full model = 7629**

**1354 missing depression (50y) data**

**1355 missing depression (50y) data**

**(Dep 50y)**

**Prospective ^e^ full model = 6288**

**Prospective ^d^ full model = 6275**

**CWP (45y):** Chronic widespread pain aged 45 years; **Dep (45y):** Depression aged 45 years; **CLBP (45y):** Chronic low back pain aged 45 years; **Dep (50y):** Depression aged 50 years; **^a^ Source:** Maddock, J., Berry, D. J., Geoffroy, M-C., Power, C. & Hypponen. (2013). Vitamin D and common mental disorders in mid-life: Cross-sectional and prospective findings. *Clinical Nutrition, 32*, 758-764. doi:10.1016/j.clnu.2013.01.006; **^b^** Depression and chronic widespread pain aged 45 years; **^c^** Depression and chronic low back pain aged 45 years; **^d^** chronic widespread pain aged 45 years and depression aged 50 years; **^e^** chronic low back pain aged 45 years and depression aged 50 years.

**Supplementary Figure 1. 1958 British birth cohort participant numbers and sample selection for the present study**

This figure demonstrates the process of deriving eligible chronic widespread and low back pain samples for the cross-sectional and prospective analyses with depression at ages 45 and 50 years.
