# Supplementary material for: Do Chronic Low Back Pain and Chronic Widespread Pain differ in their association with Depression Symptoms in the 1958 British Cohort?
Source: Pain Med. 2022 Nov 4;24(6):644–51. doi: 10.1093/pm/pnac170 (PMC10233498; doi:10.1093/pm/pnac170)
Supplement: pnac170_Supplementary_Data [file pnac170_supplementary_data.zip › pnac170_Supplementary_Data/Supplementary Table 1.docx]

| **Supplementary Table 1. Sociodemographic, lifestyle, and health characteristics of the 1958BC sample, and prevalence in the chronic pain and depression conditions.** | | | | | |
| --- | --- | --- | --- | --- | --- |
|  | **N (%)^a^** | **CWP 45 (%)** | **CLBP 45 (%)** | **DEP 45 (%)** | **DEP 50 (%)** |
| **Socio-demographic factors** |  |  |  |  |  |
| **Gender** |  |  |  |  |  |
| Male | 4665 (49.75) | 10.5 | 11.4 | 7.1 | 8.1 |
| Female | 4712 (50.25) | 12.0 | 10.5 | 9.7 | 11.6 |
| p-vlaue^b^ |  | 0.08 | 0.07 | <0.001 | <0.001 |
| **Social class** |  |  |  |  |  |
| Professional & managerial/technical | 3776 (40.27) | 8.1 | 10.8 | 6.2 | 7.7 |
| Skilled non-manual & manual | 4848 (51.70) | 12.6 | 11.0 | 8.7 | 10.7 |
| Unskilled & other/unknown | 753 (8.03) | 18.2 | 10.6 | 17.7 | 15.7 |
| p-value^b^ |  | <0.001 | 0.89 | <0.001 | <0.001 |
| **Highest qualification** |  |  |  |  |  |
| Tertiary | 2813 (30.00) | 8.7 | 9.5 | 6.3 | 7.8 |
| Secondary | 4964 (52.94) | 11.5 | 11.1 | 8.0 | 10.3 |
| No qualifications | 1299 (13.85) | 15.9 | 13.0 | 13.5 | 13.1 |
| Missing^c^ | 301 (3.21) | 3.2 | 3.5 | 4.6 | 2.9 |
| p-value^b^ |  | <0.001 | <0.001 | <0.001 | <0.001 |
| **Bill payment difficulty** |  |  |  |  |  |
| Minor/no difficulty | 5705 (60.84) | 9.3 | 11.3 | 5.9 | 7.9 |
| Difficulty | 2651 (28.27) | 16.5 | 12.9 | 10.7 | 12.5 |
| Great difficulty | 284 (3.03) | 28.9 | 12.3 | 26.1 | 28.2 |
| Missing^c^ | 737 (7.86) | 0.3 | 0.4 | 11.6 | 6.8 |
| p-value^b^ |  | <0.001 | 0.08 | <0.001 | <0.001 |
| **Separation status** |  |  |  |  |  |
| No | 8370 (89.26) | 11.4 | 11.0 | 8.0 | 9.9 |
| Yes | 630 (6.72) | 10.6 | 11.1 | 12.4 | 8.7 |
| Missing^c^ | 377 (4.02) | 3.1 | 2.9 | 5.6 | 4.2 |
| p-value^b^ |  | 0.92 | 0.63 | <0.001 | 0.83 |
| **Lifestyle factors** |  |  |  |  |  |
| **Smoking status** |  |  |  |  |  |
| Never | 4156 (44.32) | 9.4 | 9.9 | 7.3 | 8.7 |
| Ex/occasional smoker | 2721 (29.02) | 11.8 | 11.4 | 7.4 | 9.2 |
| Smoker | 2202 (23.48) | 14.1 | 12.2 | 11.4 | 12.9 |
| Missing^c^ | 298 (3.18) | 3.1 | 3.5 | 4.6 | 2.9 |
| p-value^b^ |  | <0.001 | <0.001 | <0.001 | <0.001 |
| **Physical activity** |  |  |  |  |  |
| 4 x per month or more | 5993 (63.91) | 10.5 | 10.7 | 7.1 | 9.0 |
| 3 x per month or less | 3085 (32.89) | 12.6 | 11.2 | 10.5 | 11.6 |
| Missing^c^ | 299 (3.19) | 3.2 | 3.5 | 4.7 | 3.0 |
| p-value^b^ |  | <0.001 | 0.45 | <0.001 | <0.001 |
| **Screen time for leisure** |  |  |  |  |  |
| Non-sedentary (≤ 4 hours) | 7950 (84.78) | 10.8 | 11.1 | 8.0 | 9.5 |
| Sedentary (> 4 hours) | 950 (10.13) | 15.4 | 11.8 | 10.4 | 12.3 |
| Missing^c^ | 477 (5.09) | 4.6 | 3.2 | 7.2 | 5.6 |
| p-value^b^ |  | <0.001 | 0.40 | 0.01 | <0.001 |
| **Alcohol intake** |  |  |  |  |  |
| Never/rarely | 2001 (21.34) | 16.6 | 11.1 | 13.4 | 13.7 |
| Light/moderate | 4890 (52.15) | 9.8 | 10.7 | 6.9 | 8.5 |
| Heavy | 2422 (25.83) | 9.8 | 11.2 | 7.4 | 9.5 |
| Missing^c^ | 64 (0.68) | 0.5 | 0.5 | 0.4 | 0.4 |
| p-value^b^ |  | <0.001 | 0.70 | <0.001 | <0.001 |
| **Social contact** |  |  |  |  |  |
| Multiple times per month | 7236 (77.17) | 12.3 | 11.9 | 7.6 | 9.3 |
| Once monthly or less | 1401 (14.94) | 11.6 | 11.4 | 10.6 | 13.6 |
| Missing^c^ | 740 (7.89) | 0.1 | 0.2 | 11.8 | 7.1 |
| p-value^b^ |  | 0.48 | 0.53 | <0.001 | <0.001 |
| **Supplementary table 1. (continued)**  **Active social group member** |  |  |  |  |  |
| Yes | 3633 (38.74) | 10.5 | 11.8 | 6.0 | 7.7 |
| No | 4980 (53.11) | 13.4 | 11.8 | 9.5 | 11.6 |
| Missing^c^ | 764 (8.15) | 0.5 | 0.6 | 12.4 | 7.2 |
| p-value^b^ |  | <0.001 | 0.92 | <0.001 | <0.001 |
| **Health status** |  |  |  |  |  |
| **General health** |  |  |  |  |  |
| Excellent | 1551 (16.54) | 3.7 | 7.2 | 3.2 | 5.3 |
| Good | 5810 (61.96) | 9.5 | 11.5 | 6.5 | 7.9 |
| Fair / poor | 1754 (18.71) | 24.7 | 13.2 | 19.0 | 20.5 |
| Missing^c^ | 262 (2.79) | 1.5 | 1.1 | 3.4 | 2.5 |
| p-value^b^ |  | <0.001 | <0.001 | <0.001 | <0.001 |
| **Body Mass Index** |  |  |  |  |  |
| Normal | 3116 (33.23) | 10.0 | 10.7 | 8.3 | 7.6 |
| Underweight | 51 (0.54) | 13.6 | 9.1 | 17.6 | 20.5 |
| Overweight | 3796 (40.48) | 12.0 | 12.8 | 7.4 | 6.9 |
| Obese | 2247 (23.96) | 15.9 | 12.3 | 10.0 | 9.1 |
| Missing^c^ | 167 (1.78) | 18.2 | 14.3 | 11.9 | 11.6 |
| p-value^b^ |  | <0.001 | 0.06 | <0.001 | <0.001 |
| **CWP 45:** Chronic widespread pain aged 45 years; **CLBP 45:** Chronic low back pain aged 45 years; **DEP 45:** Depression aged 45 years; **DEP 50:** Depression aged 50 years; **^a^ N** = 9377; **^b^** Chi test P-value, excluding participants with unknown values;  **^c^** Indicates percentage of phenotype missing socio-demographic, lifestyle or health information. | | | | | |
|  |  |  |  |  |  |
|  |  |  |  |  |  |
|  |  |  |  |  |  |
